# Dietary protein defends lean mass and maintains the metabolic benefits of glucagon receptor agonism in mice

**DOI:** 10.1016/j.molmet.2024.102024

**Published:** 2024-09-03

**Authors:** Tatiana Lopes, David CD. Hope, Jose M. Ramos-Pittol, Anna Curtis, Jed V. Shrewsbury, Iona Davies, Zijing Zhou, Alessandro Sardini, James S. Minnion, Dirk Dormann, Gavin A. Bewick, Kevin G. Murphy, David Carling, Stephen R. Bloom, Tricia MM. Tan, Bryn M. Owen

**Affiliations:** 1 Section of Investigative Medicine, Imperial College London, United Kingdom; 2 Institute of Biochemistry, University of Innsbruck, Austria; 3 MRC Laboratory of Medical Sciences, London, United Kingdom; 4 Institute of Clinical Sciences, Imperial College London, London, United Kingdom; 5 Diabetes and Obesity Theme, School of Cardiovascular and Metabolic Medicine and Sciences, Faculty of Medicine, Kings College London and Diabetes Endocrinology and Obesity Clinical Academic Partnership Kings Health Partners, UK

**Keywords:** Glucagon, Obesity, MAFLD, Drug, Side-effect, Lean mass, Muscle mass

## Abstract

**Objective:**

Glucagon has long been proposed as a component of multi-agonist obesity therapeutics due to its ability to induce energy expenditure and cause weight loss. However, chronic glucagon-receptor agonism has been associated with a reduction in circulating amino acids and loss of lean mass. Importantly, it is currently not known whether the metabolic benefits of glucagon can be maintained under contexts that allow the defence of lean mass.

**Methods:**

We investigate the metabolic effects of the long-acting glucagon receptor agonist, G108, when administered to obese mice at low-doses, and with dietary protein supplementation.

**Results:**

Dietary protein supplementation can only fully defend lean mass at a low dose of G108 that is sub-anorectic and does not reduce fat mass. However, in this context, G108 is still highly effective at improving glucose tolerance and reducing liver fat in obese mice. Mechanistically, liver RNA-Seq analysis reveals that dietary protein supplementation defends anabolic processes in low-dose G108-treated mice, and its effects on treatment-relevant glucose and lipid pathways are preserved.

**Conclusion:**

Glucagon-mediated energy expenditure and weight loss may be mechanistically coupled to hypoaminocidemia and lean mass loss. However, our data suggest that glucagon can treat MAFLD at doses which allow full defence of lean mass given sufficient dietary protein intake. Therefore, proportionate glucagon therapy may be safe and effective in targeting hepatocytes and improving in glycaemia and liver fat.

## Highlights

• Glucagon analogue, G108, improves metabolic parameters independent of weight loss.
• Dietary protein supplementation fully defends lean mass at low doses of G108.
• The metabolic benefits of G108 are not affected by dietary protein supplementation.
• Liver RNA-seq analysis provides mechanistic insight in to G108-action.

## Graphical abstract

Low dose glucagon agonism stimulates amino acid catabolism and lipid metabolism in liver. A high protein diet prevents the consequences of hypoaminoacidemia without affecting beneficial effects on MAFLD.

**Figure:**
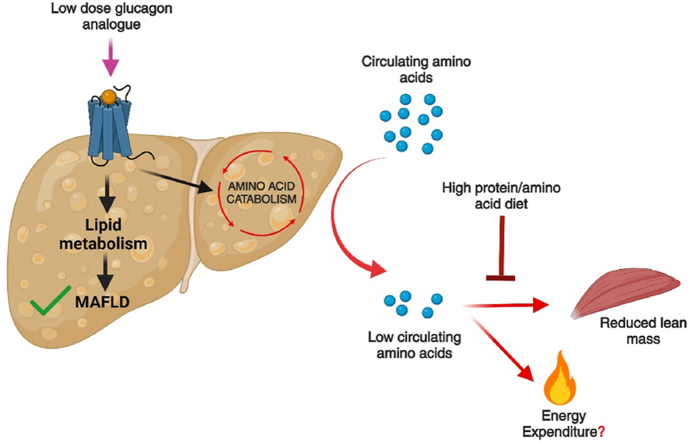

## 1 Introduction

Glucagon is a 29 amino acid peptide-hormone that is secreted from pancreatic alpha-cells and stimulates hepatic glucose production [1]. It also stimulates the catabolism of lipids and amino acids in liver [2]. In this way, glucagon physiologically contributes to the maintenance of blood glucose during fasting [1]. It is also part of the physiological liver-pancreatic alpha cell axis in which amino acids and glucagon are involved in a reciprocal feedback loop, with amino acids from food stimulating glucagon secretion and hence amino acid catabolism [3].

In pharmacological settings, glucagon can reduce body weight by suppressing appetite and stimulating energy expenditure [2,[4], [5], [6], [7]]. Direct glucagon signalling in the liver also promotes lipid catabolism, thus improving symptoms of MAFLD [7,8]. Somewhat counterintuitively, chronic glucagon receptor agonism also improves glycemia [[6], [7], [8], [9]]. Together, these functions have resulted in the investigation of glucagon analogues as metabolic-disease treatments, often in combination with other hormone analogues such as GLP-1 analogues [2,10]. Examples include the GLP-1/GIP/glucagon triple-agonist Retatrutide and the GLP-1/glucagon co-agonist Survodutide [11,12]. However, glucagon receptor agonism upregulates pathways of hepatic amino acid catabolism and reduces plasma amino acids [8,[13], [14], [15]]. This has been associated with a loss of muscle mass in mice [7]. Although effects of glucagon-targeted multi-agonists on lean mass in humans is unclear, there are concerns about lean mass loss as a potential unwanted side effect. It is therefore important to determine treatment regimens that prevent lean mass loss in the context of glucagon receptor agonism.

We have previously shown that daily treatment with G108, a long-acting glucagon analogue, at a dose of 7.5 nmol/kg, causes weight loss in obese mice without affecting food intake [7]. This effect was attributed to a systemic induction of energy expenditure in response to hypoaminoacidemia, and was associated with a reduction in lean mass [7]. Indeed, dietary protein supplementation partially protected muscle mass in G108-treated mice, and completely prevented fat mass loss [7]. Despite this, metabolic parameters including glucose tolerance and hepatic fat content were still improved by G108 with protein supplementation [7]. We therefore concluded that glucagon receptor agonism remains a promising candidate for metabolic disease treatment. However, our protein supplementation strategy only partially defended lean mass [7]. It therefore remained to be seen whether some degree of lean mass loss is a pre-requisite for the full metabolic benefits of G108 in obese mice.

Here, we investigate low doses of G108 in combination with protein supplementation. We find that dietary protein supplementation can only fully defend lean mass at a dose of G108 that is sub-anorectic and does not reduce fat mass. However, in this context, G108 still profoundly improves liver fat and glycemia in obese mice. RNA-seq analysis of liver samples reveal that protein supplementation defends anabolic processes but does not interfere with clinically-beneficial G108-effects. These findings may therefore have translational implications for the use of GCGR-targeted multi-agonists for the treatment of obesity and metabolic disease.

## 2 Materials and methods

### 2.1 Animal studies

All procedures were conducted on male mice under authority of the U.K Animals (Scientific Procedures) Act, 1986, and approved by the Animal Welfare Ethical Review Body of Imperial College London. A preliminary study had established no sexual dimorphism in response to G108. Animals were housed under standard conditions in individually ventilated cages with free access to food and water, environmental enrichment, and wood-chip bedding. Lights-on 07:00 h, lights off 19:00 h. Mice were C57BL6/J strain and obtained from Charles River UK. G108 [7] is a custom peptide obtained from WuXi AppTec, China. It was diluted in HPLC grade water prior to subcutaneous administration. Diets were from Research Diets inc. Lean 6-week-old C57BL/6 mice were provided ad libitum a 60 kcal% high fat diet (diet D12492) for 6 weeks. They were then transferred to SP or HO diets for two weeks prior to the start of the G108 treatments. The cohort of mice were individually housed and randomly divided into treatment groups; the high fat, standard protein diet (SP diet) (D08091803) and high fat, high protein diet (HP diet) (D08091801). All mice had ad libitum access to water and their allocated diets. Mice were subcutaneously administered with their respective treatments at 9 am each day for 22 days. Food intake and body weight were measured using the same weighing scale each morning prior to vehicle or G108 administration. EchoMRI (EchoMRI −100H, Houston, Tx.) was carried out at baseline, day 13 and day 21. A glucose tolerance test was carried out on day 15 of the study following a 5 h fast. Forelimb grip strength testing was performed at baseline and day 21 of the study. Following a 4 h fast on day 22 all mice were culled, with tissues snap frozen in liquid nitrogen prior to storage at −80 °C. Terminal blood was also collected and transferred on ice immediately for centrifugation and storage at −80 °C.

### 2.2 Grip strength

A grip strength meter (Bioseb, France) was used to measure grip strength. Mice were individually lowered on to a grip strength bar prior to gentle retraction of the tail until grip release. The maximum force, measured in g was recorded. Four consecutive measurements were made with a 1 min rest interval. An average of measurements was taken for each timepoint prior to subsequent analysis.

### 2.3 Glucose tolerance tests

For glucose tolerance tests, following a 5 h fast and administration of peptide or vehicle treatment, mice were administered with glucose by intraperitoneal injection at a dose of 2 g/kg body weight. Tail vein blood samples were taken at baseline, 20, 40, 60 and 90 min and glucose measured (mmol/L) using a handheld GlucoRx monitor (GlucoRx, UK).

### 2.4 Liver histology

Frozen liver samples were sectioned and at 8–10 mm and fixed in formalin. Sections were stained with Oil Red O according to standard protocols, prior to imaging. Representative samples are shown. Quantification of oil red staining was performed using the QuPath image analysis software (version 0.4.3). Large tissue regions (annotations) were manually created to avoid damaged or folded parts of the tissue sections. All annotations were then further processed automatically to exclude blood vessels or other cavities inside the tissue. Oil red O staining in the tissue was detected with a pixel classifier that had been trained on representative images [16].

### 2.5 RNA-sequencing and analysis

RNA was extracted from liver using 1 mL TRIzol added to samples prior to homogenisation within a mechanical homogeniser for 2–3 min 200ul chloroform was added to each individual sample prior to gentle shaking and centrifugation at 16000×*g* for 15 min at 4 °C. The supernatant was decanted into separate Eppendorf tubes and a 1:1 mixture of supernatant to 70% iso-propanol was made prior to further centrifugation at 16000×*g* for 15 min. The RNA pellet was washed three times with 70% ethanol and resuspended in sterile RNase-free water. RNA was quantified using a NanoDrop spectrophotometer. RNA sequencing procedures were performed by NovoGene UK. Sequencing libraries were prepared using the NGS RNA Library Prep Set (PT042, Novogene). RNAseq was performed on the Novaseq 6000 platform (Illumina). Quality control on the sequence reads from the raw FASTQ files was done with FastQC (v0.11.8). Reads were aligned to the reference genome GRCm38_snp_tran using HISAT2 (v2.0.5) [17]. Readcounts were generated using the Rsubread FeatureCounts module (v2.0.0) with the *Mus musculus* GRCm38.102 gtf file as annotation [18]. Transcripts per million (TPM) were calculated based on the number of reads count mapped to each gene over the exon length of the respective gene x 1 million, divided by the total number of mapped reads. Differential expressed (DE) genes were determined with DESeq2 (v 1.36.0) [19]. Shrinkage of effect size was performed on Deseq2 results using the apeglm method through the function lfcShrink [20]. Principal component analysis was performed on TPM values using the R function prcomp with the option “scale. = TRUE”, and plotted on ggplot2 [21]. Rsubread, Deseq2 and lfcShrink are Bioconductor packages (Release 3.19) and were executed in RStudio 2024.04.0 Build 735 under R 4.4.0. Raw reads and TPM values from RNA sequencing experiments performed during this study can be found on the Gene Expression Omnibus [22] under accession number GSE273658.

### 2.6 Statistical analysis

All statistical analysis were conducted in GraphPad Prism 9.0.0 (GraphPad Software). Statistical significance levels and n numbers are reported in figure legends in addition to dispersion represented as mean and SEM. Significance is defined as p values below 0.05. Assumptions for parametric tests included normal distribution of datasets.

## 3 Results

### 3.1 Establishing a metabolically-active low dose of G108

We have previously developed a long-acting glucagon analogue, G108. It has over a 120-fold selectivity factor for the GCGR over the GLP-1R, which is similar to native glucagon. In a chronic setting, we have previously shown that G108 causes weight loss and improves metabolic parameters at a dose of 7.5 nmol/kg. However, it also causes muscle wasting at this dose, and protein supplementation mitigates, but does not prevent this [7]. We therefore asked here whether protein supplementation would be fully effective at defending lean mass at lower, but still therapeutically-effective, doses of G108. To answer this question, diet-induced obese mice were randomized to receive vehicle, G108 at 5 nmol/kg, or G108 at 2.5 nmol/kg plus or minus dietary protein supplementation, for 22-days.

**Figure 1:**
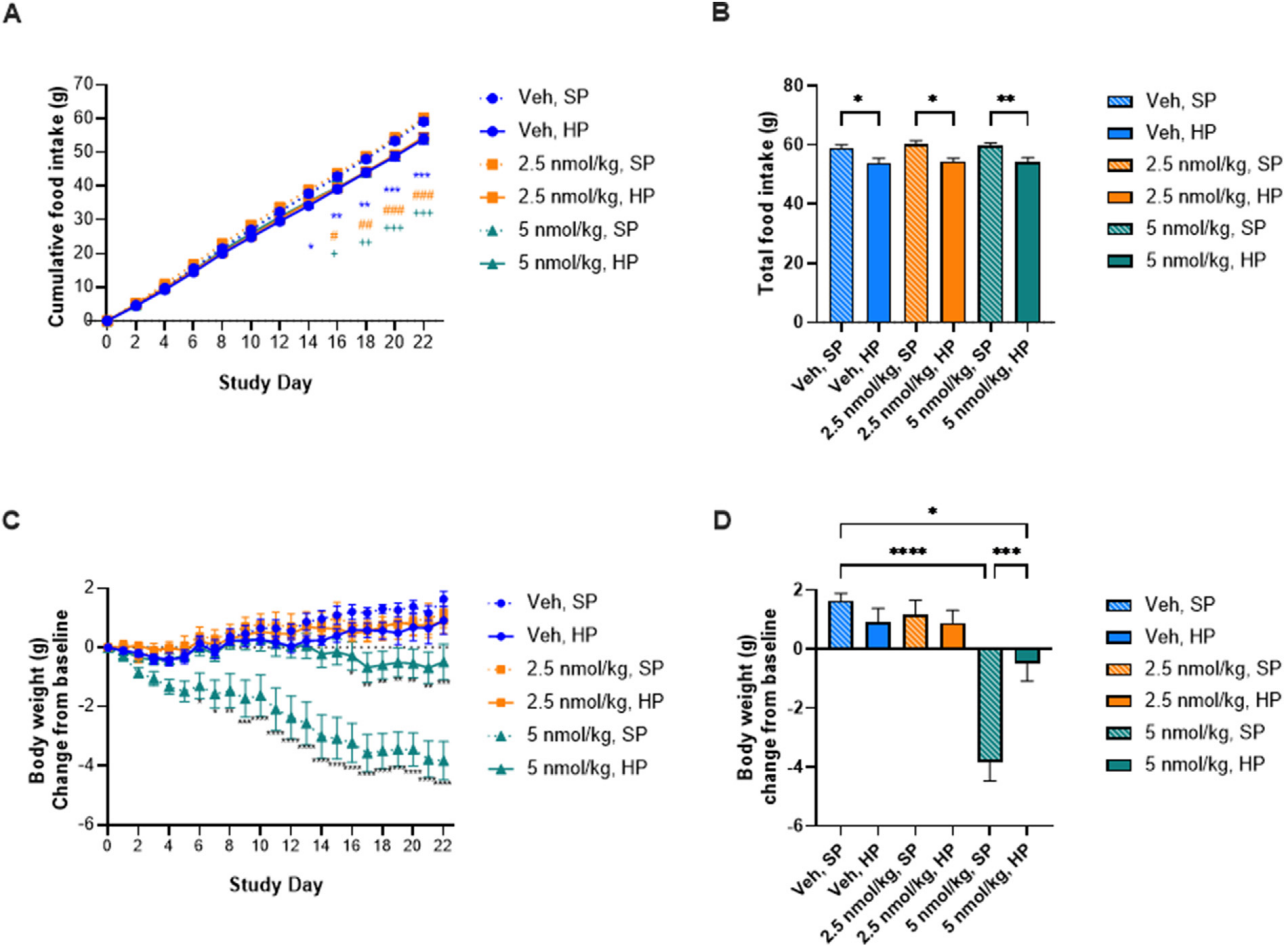
*Establishing a low dose of G108*. (A and B) Food intake. (C and D) Body weight.

As expected, protein supplementation itself slightly reduced food intake (Figure 1A). However, G108 did not affect food intake at 2.5 nmol/kg or 5 nmol/kg (Figure 1B). As we have seen previously with a dose of 7.5 nmol/kg [7], G108 at 5 nmol/kg caused sustained weight loss in obese mice without protein supplementation (Figure 1B). Also consistent with our previous observations [7], G108-mediated weight loss was almost completely abolished by dietary protein supplementation (Figure 1B). At the even lower dose of G108 (2.5 nmol/kg), we did not observe statistically significant weight-loss on either diet (Figure 1B). Together, these data confirm that protein supplementation prevents G108-mediated weight loss in the sub-anorectic range, and establish a threshold-dose for weight-loss on a standard diet.

### 3.2 Dietary protein supplementation fully preserves lean mass in low-dose G108 treated mice

**Figure 2:**
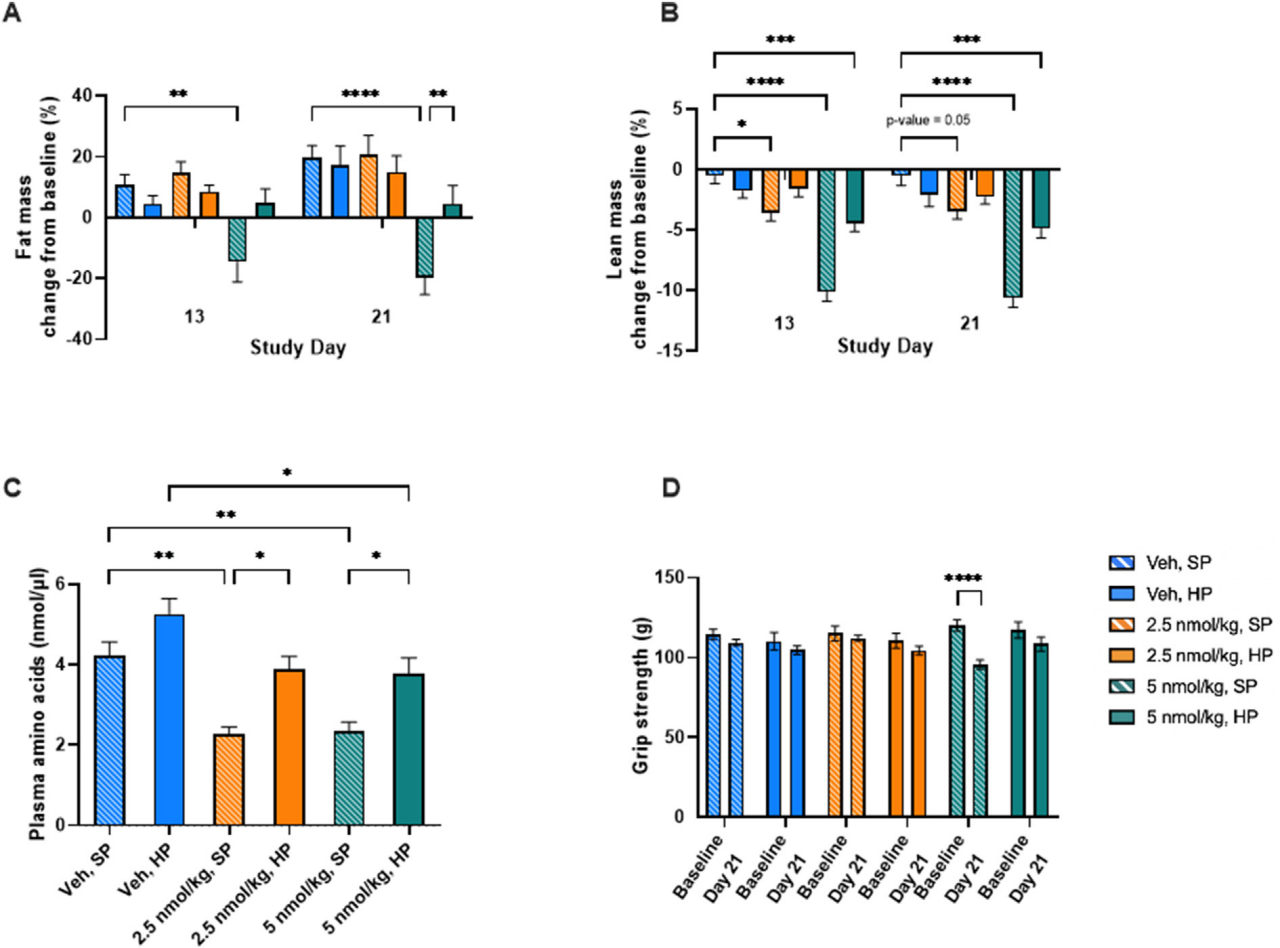
*Dietary protein supplementation fully preserves lean mass in low-dose G108 treated mice.* (A) Fat mass. (B) Lean mass. (C) Plasma total amino acids. (D) Grip strength.

We next assessed fat and lean mass using EcoMRI in DIO mice treated with low doses of G108 with and without dietary protein supplementation. As we have seen previously with a dose of 7.5 nmol/kg [7], G108 at 5 nmol/kg caused a significant reduction in fat mass in obese mice without protein supplementation (Figure 2A). Also consistent with our previous observations [7], the G108-mediated reduction in fat mass was almost completely abolished by dietary protein supplementation (Figure 2A). At 2.5 nmol/kg, G108 had no effect on fat mass either on standard diet or with protein supplementation (Figure 2A).

There was a highly significant reduction in lean mass in mice treated with 5 nmol/kg G108 without protein supplementation (Figure 2B). As we saw previously with 7.5 nmol/kg G108 [7], this effect was blunted, but not eliminated, by protein supplementation (Figure 2B). At 2.5 nmol/kg, and without protein supplementation, G108 caused a small but statistically significant reduction in lean mass (Figure 2B). However, protein supplementation at this dose (2.5 nmol/kg) of G108 was fully effective at protecting lean mass (Figure 2B). Terminal plasma amino acid analysis following a 4 h fast demonstrated that our dietary protein intervention was largely effective at rescuing G108 mediated hypoaminoacidemia (Figure 2C) Functional grip strength reduced over the course of the study only in mice receiving 5 nmol/kg G108 without protein supplementation (Figure 2D). Together, these data show that lean mass and functional muscle mass can be fully defended by protein supplementation in mice receiving low dose G108.

### 3.3 Low-dose G108 improves glycemia and liver steatosis in the presence of protein supplementation

**Figure 3:**
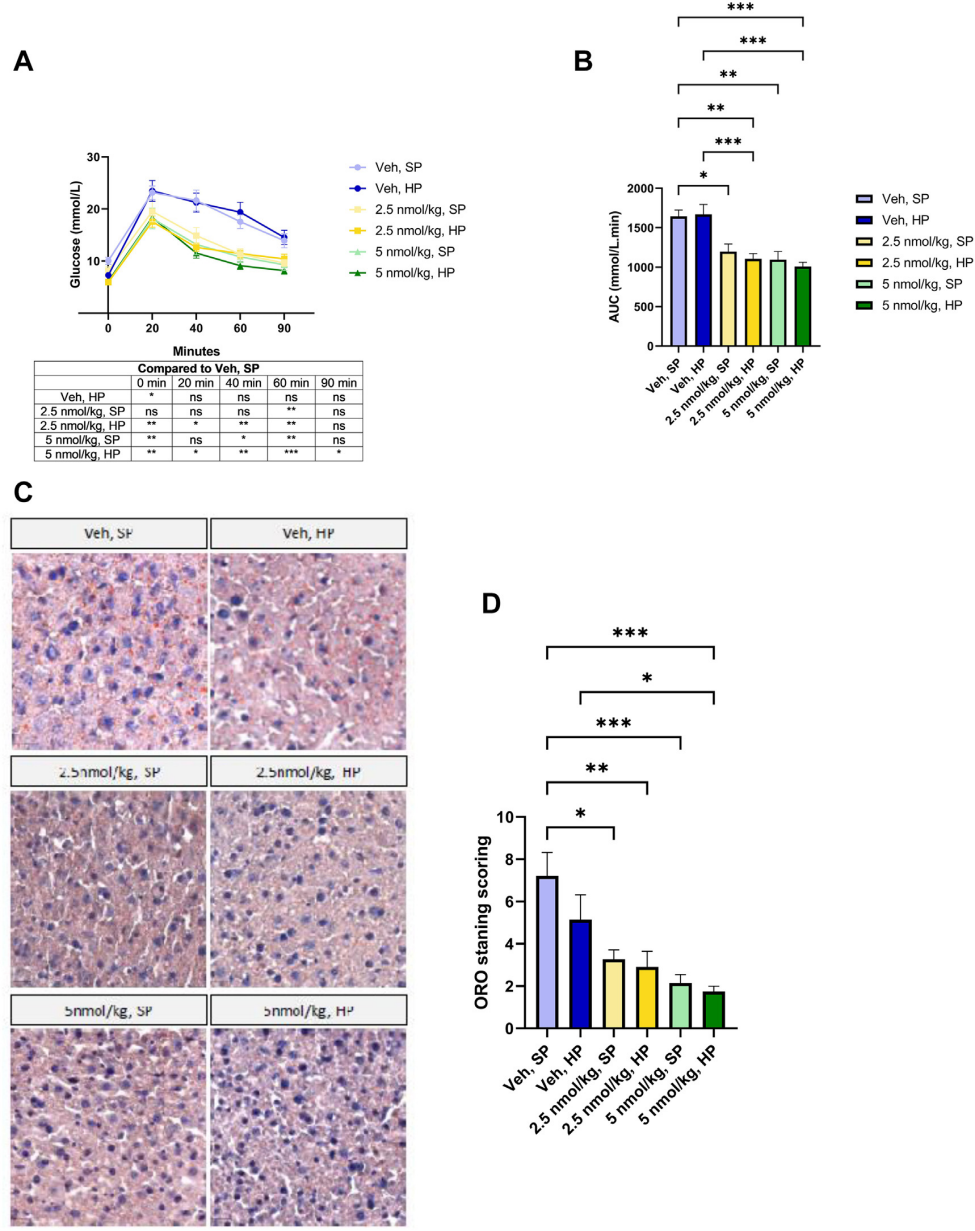
*Low-dose G108 improves glycemia and liver steatosis in the presence of protein supplementation.* (A) Glucose tolerance test and (B) area under the curve analysis. (C) Representative images and (D) quantification of Oil Red O staining of liver sections. (For interpretation of the references to color in this figure legend, the reader is referred to the Web version of this article).

We next assessed the clinically important question of how G108 affects metabolic parameters at low doses with and without protein supplementation. G108-treated mice had a marked improvement in glucose tolerance at both doses, and both with and without protein supplementation (Figure 3A,B). Indeed, while there were some treatment-specific differences in statistical significance at individual time-points (Figure 3A), the 2.5 nmol/kg and 5 nmol/kg dose of G108 were broadly equally effective in reducing glucose area under the curve (Figure 3B). The addition of dietary protein had either no effect on G108-mediated improvement in glucose tolerance, or even marginally enhanced it (Figure 3A,B). G108 was still highly effective at reducing hepatic fat content at both doses (Figure 3C,D). Similar to the glucose tolerance test, there was no indication that dietary protein supplementation hindered the ability of G108 to clear liver fat (Figure 3C,D). These data demonstrate that the metabolic benefits of G108 can be uncoupled from a reduction in lean mass by the use of a low dose and dietary protein supplementation.

### 3.4 Anabolic processes, and therapeutically relevant G108-stimulated pathways are preserved under protein supplementation

**Figure 4:**
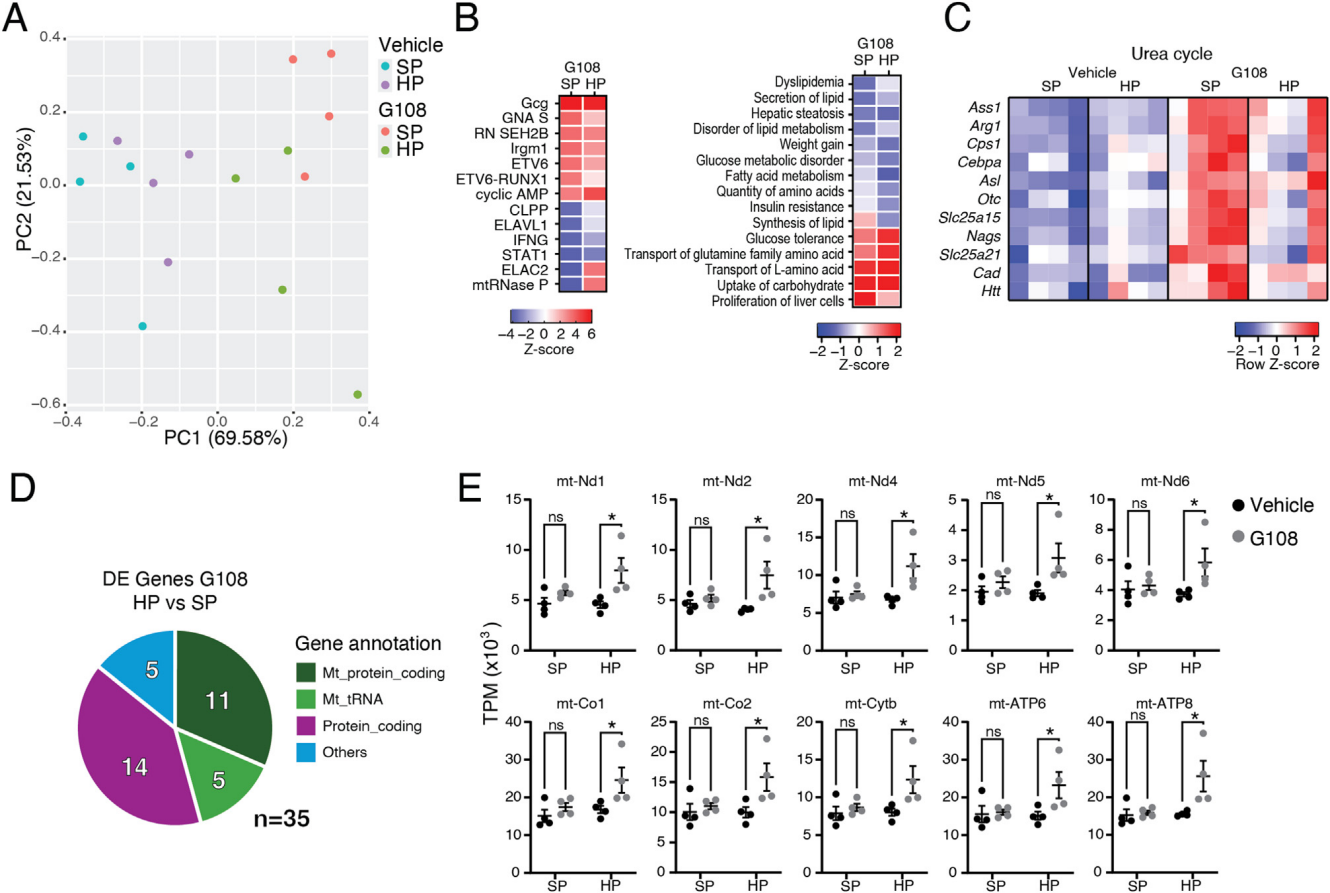
*Therapeutically relevant G108-stimulated metabolic pathways are preserved under protein supplementation but mitochondrial transcripts are cooperatively enhanced.* (A) Principal component analysis using full transcriptomes. (B) Ingenuity Pathway Analysis ‘upstream regulators’ (right) and ‘disease processes’ (Left) in genes affected by G108 treatment. (C) Expression of genes in the Urea Cycle pathway. (D) Annotation of differentially expressed genes in G108 treated mice with vs without protein supplementation. (E) Mitochondrial transcripts that are elevated by protein supplementation in G108 treated mice.

In order to gain mechanistic insight into the effects of dietary protein supplementation during low-dose G108 treatment, we conducted RNA-sequencing of liver tissue. Initial analysis demonstrated that G108 at a dose of 2.5 nmol/kg on standard chow or high protein diet showed clear separation compared to vehicle on principal component analysis (Figure 4A). Indeed, across the entire experiment, approximately 70% of the variance in gene expression can be captured on the first dimension of PCA where G108 treatment is the defining factor (Figure 4A). By contrast, protein supplementation made only a minor contribution (Figure 4A). Importantly, effects of G108 on therapeutically relevant pathways associated with known glucagon action were broadly maintained under dietary protein supplementation (Figure 4B,C). Specifically, IPA analysis demonstrated that ‘glucagon’ was the most prominent upstream regulator in G108 treated mice, despite the very low dose used here, and regardless of dietary protein content (Figure 4B). Furthermore, G108 at a dose of 2.5 nmol/kg on both standard chow and high protein diet increased the expression of transcripts associated with amino acid transport, carbohydrate uptake, and glucose tolerance (Figure 4B). Conversely, genes associated with dysglycemia, hepatic steatosis, fatty acid metabolism, and secretion of lipids were decreased by G108 treatment at 2.5 nmol/kg on both standard chow and high-protein diet (Figure 4B). Finally, genes in the urea cycle pathway, which have been previously shown to be induced by glucagon in the liver [7,23], were induced by G108 treatment at 2.5 nmol/kg both on standard chow or high protein diet, albeit to a lesser extent in the latter (Figure 4C). Together, these results confirm that G108 affects liver gene expression pathways similarly to other glucagon analogues, despite being used here at a sub-anorectic dose that does not affect fat mass. The effect of G108 on these pathways is broadly maintained by dietary protein supplementation.

Next, we focused on differentially expressed genes driven by protein supplementation in G108 treated mice. Of the few differentially expressed genes, almost half were mitochondrial protein-coding and tRNA transcripts (Figure 4D). These genes showed a cooperative induction by G108 and dietary protein supplementation (Figure 4E), and are involved in mitochondrial respiration, suggesting that hepatic mitochondrial biosynthesis can be enhanced in G108-treated mice by simply maintaining sufficient dietary protein consumption. Many of the other induced genes (Figure 4D) were members of the Major Urinary Protein family, abundantly-synthesized secreted proteins in mouse, which were defended by dietary protein supplementation in G108 treated animals. Therefore, these data demonstrate that in G108 treated animals, anabolic processes in liver are protected by protein supplementation.

## 4 Discussion

We have shown that dietary protein supplementation can only fully defend lean mass at a dose of G108 which is both sub-anorectic, and does not reduce fat mass. However, low dose G108 is also still highly effective at improving glycemia and reducing liver fat in obese mice fully protected against lean mass loss by dietary protein. Indeed, our liver RNA-Seq analysis confirm that therapeutically relevant lipid and glucose-regulatory pathways are maintained under protein supplementation in G108 treated mice, and that anabolic pathways are defended.

We observed a striking effect of chronic, low dose, G108 on glucose tolerance in obese mice. This is intriguing, as glucagon action would be predicted to worsen glucose tolerance. Indeed, glucagon antagonists have been proposed as potential anti-hyperglycaemic agents in type 2 diabetes mellitus [24]. The precise mechanisms through which chronic pharmacological glucagon administration may improve glycemia remains unclear. It is possible that the potent beneficial effects of glucagon in MAFLD may be due to improved insulin sensitivity, glucose handling and metabolic flexibility [25]. Interestingly, chronic G108 increased the expression of several gluconeogenic and glycogenolytic genes in the liver, further complicating understanding of the metabolic changes observed. But interpreting changes in gene expression is challenging without concurrent information on substrate availability and metabolite flux. It is also possible that central, or other peripheral effects, of G108 signalling may also contribute to enhanced glucose tolerance. However, the data presented here are consistent with an interpretation that glucagon signalling at the level of hepatocytes may contribute to improving glucose tolerance in the context of obesity and MAFLD.

Our RNA-Seq analysis provide invaluable insight into the mechanisms of G108 action. Our data confirm glucagon-mediated induction of urea cycle genes, even at a sub-anorectic dose that does not affect fat mass. Our data also indicate two major mechanisms of G108 function on liver. First, direct signalling to lipid and glucose pathways which are largely unaffected by dietary protein supplementation. Second, effects on mitochondrial biosynthesis and anabolic pathways which are enhanced by protein supplementation and are therefore likely an indirect interaction between G108 and dietary protein intake. It is noteworthy that hepatic mitochondria play a pivotal role in fatty acid oxidation, lipogenesis and gluconeogenesis. Indeed, increased mitochondrial metabolism in the liver may enhance lipid clearance and contribute to glucose tolerance in these models [26].

Glucagon has long been proposed as a component of multi-agonist anti-obesity therapeutics due to its ability to induce energy expenditure and cause weight loss [6,27]. However, our recent studies have suggested that hypoaminoacidemia is one potential contributing factor to these effects [7,28]. The data presented here support this conclusion. Indeed, our findings suggest that in the absence of sufficient dietary protein intake, a reduction in lean mass occurs at a low dose of G108 that does not affect food intake or reduce fat mass. However, dietary protein supplementation used here can fully defend lean mass at this dose, and we show that it is still highly effective at improving liver fat and glycemia in obese mice. Our data therefore suggest that very low doses of glucagon may essentially act as hepatocyte-targeting therapeutics to improve glucose tolerance and reduce liver fat without inducing energy expenditure, weight loss, or appetite suppression.

It is crucial to establish how pre-clinical findings on glucagon pharmacotherapy might translate to humans. Our work in mice has demonstrated how different doses of G108 can have very different effects on metabolism. For example, 2.5 nmol/kg G108 is metabolically effective in MAFLD but 5 nmol/kg G108 is required to reduce body fat, and 10 nmol/kg is required to suppress appetite. Our data also show that dietary protein content can dramatically influence some aspects of chronic glucagon biology in mice, and others have shown that hepatic steatosis can influence glucagon sensitivity [14]. Therefore, understanding glucagon action in humans requires careful consideration of dose, glucagon (and insulin) sensitivity, and dietary protein content. Very high doses of glucagon can activate the brown adipose tissue in mice, a mechanism of action that is likely of limited therapeutic potential in humans [27]. However, direct glucagon signalling at the level of the hepatocyte is likely translatable, and evidence is emerging to confirm the beneficial hepatic effects of GCGR-targeted multi-agonists in humans [29]. Finally, it is academically interesting to speculate about the systemic effects of glucagon-induced hypoaminoacidemia. For example, consideration could be given to the effects of this induced hypoaminoacidemia on chronic inflammation, longevity, muscle turnover, tumorigenesis, or pathophysiological CNS protein deposition. These possibilities, of course, require considerable thought before pre-clinical testing. Ultimately, however, our findings will continue to re-frame the proposed used of glucagon in so-called multi-agonist metabolic disease therapeutics. Specifically, we propose targeting low-dose glucagon action to improving MAFLD, rather than inducing weight loss.

## CRediT authorship contribution statement

**Tatiana Lopes:** Writing – review & editing, Methodology, Investigation, Formal analysis. **David CD. Hope:** Writing – review & editing, Writing – original draft, Methodology, Investigation, Formal analysis, Data curation, Conceptualization. **Jose M. Ramos-Pittol:** Writing – review & editing, Writing – original draft, Methodology, Formal analysis, Data curation. **Anna Curtis:** Investigation. **Jed V. Shrewsbury:** Investigation. **Iona Davies:** Investigation. **Zijing Zhou:** Investigation. **Alessandro Sardini:** Investigation. **James S. Minnion:** Investigation. **Dirk Dormann:** Methodology. **Gavin A. Bewick:** Methodology. **Keving G. Murphy:** Writing – review & editing, Supervision. **David Carling:** Supervision. **Stephen R. Bloom:** Supervision, Conceptualization. **Tricia MM. Tan:** Writing – review & editing, Supervision, Conceptualization. **Bryn M. Owen:** Writing – review & editing, Writing – original draft, Supervision, Project administration, Investigation, Formal analysis, Conceptualization.

## Declaration of competing interest

TMMT is a consultant and shareholder in Zihipp/Metsera. JSM and SRB are employees and shareholders in Zihipp/Metsera, which is developing gut hormone analogues for treatment of metabolic disease.

## Data Availability

Data will be made available on request.
